# The role of intraspecific competition between plants in a nursery pollination system—Comments on Villacañas de Castro and Hoffmeister 2020

**DOI:** 10.1002/ece3.6837

**Published:** 2020-10-21

**Authors:** Nicolas M. Gutiérrez, Luciano Stucchi, Javier Galeano, Luis Giménez‐Benavides

**Affiliations:** ^1^ Universidad del Pacífico Lima Perú; ^2^ Departamento de Ciencias Pontificia Universidad Católica del Perú Lima Perú; ^3^ Grupo de Sistemas Complejos Universidad Politécnica de Madrid Madrid Spain; ^4^ Departamento de Biología, Geología, Física y Química Inorgánica Universidad Rey Juan Carlos‐ESCET Madrid Spain

**Keywords:** tritrophic interactions, population dynamic modeling

## Abstract

We present comments on an article published by Villacañas de Castro and Hoffmeister (Ecology and Evolution, 10, 4220; 2020). The authors studied a tritrophic system composed of a plant, its pollinating seed predator, and a parasitoid of the latter. Their concern was whether the parasitoid modifies the interaction between the plant and its pollinator–herbivore along the mutualism–antagonism gradient, but they reduced their question to how the parasitoid impacts plant fitness. After showing that the parasitoid increases seed output of the plant by decreasing the amount of seeds consumed by the pollinating seed predator, they tested whether seed output is a good proxy for plant fitness. They argue that it is not by showing that the increased seed density has a negative impact on survival probability and flower production, likely due to plant intraspecific competition. The work presented shows careful experimentation and interesting results, but we do not share some of their conclusions. Most importantly, we believe that the net effect of the parasitoid on the plant–herbivore interaction cannot be adequately investigated by focusing on individual plant fitness. Thus, we first suggest considering the number of surviving plants up to adulthood as a proxy for population performance to address this question. Using this proxy, we show that the increase in seed output due to the parasitoid is beneficial to the plant population until its carrying capacity is achieved. Next, using a population dynamics model, we show under which particular conditions the negative effect of intraspecific competition outweighs the positive effect of seed density increase (due to parasitoid's defense). When these conditions do not hold, the role of plant intraspecific competition is basically limited to the prevention of unbounded population growth, while the parasitoid increases the plant's equilibrium density above its carrying capacity as measured when interacting only with the pollinating seed predator, thus making the system more stable.

## Graphical abstract

We comment a recent article published by Villacanas de Castro & Hoffmeister on the tritrophic system composed by Silene, its pollinating seed predator Hadena, and its parasitoid Bracon.

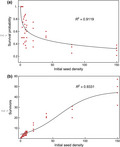

## 1 INTRODUCTION

We have read with great interest the article published by Villacañas de Castro and Hoffmeister titled *Friend or foe? A parasitic wasp shifts the cost/benefit ratio in a nursery pollination system impacting plant fitness* (Villacañas de Castro & Hoffmeister, 2020). Therein, the authors studied a tritrophic system composed of a plant, *Silene latifolia*, its pollinating seed predator, *Hadena bicruris*, and a parasitoid of the latter, *Bracon variator*. The *S. latifolia–H. bicruris* interaction is usually described as antagonistic and, in their paper, it can be read that their main concern was whether the presence of the parasitoid modifies the interaction between the plant and its herbivore along the mutualism–antagonism gradient. Thus, their stated research questions are as follows: 1) *can a natural enemy* (in this case, the parasitoid) *impact the level of seed consumption by the seed predator* and 2) *if so, what are the consequences at the level of individual plant fitness*. To answer these questions, Villacañas de Castro and Hoffmeister performed an interesting series of laboratory and greenhouse experiments and painstakingly collected data on seed predation and different proxies for individual plant fitness: seed output, germination, survival to adulthood, and lifetime flower production.

In their first experiment, they measured seed output of female *S. latifolia* plants submitted to different treatments: a) “without herbivore attack,” b) “with herbivore attack,” and c) “with herbivore attack plus parasitoids.” As a result, the authors convincingly show that the presence of *B. variator* decreases the level of seed consumption by *H. bicruris* larvae, thus increasing the net seed output of *S. latifolia*, and therefore suggesting that *B. variator* may act as a regulator in the system. This is an interesting result that agrees with their prediction and with previous results in this and other nursery pollination systems.

The authors carried out a second experiment to explore the potential effect of the increase in seed output on individual plant fitness. They set densities of 1, 2, 3, 4, 5, 6, 7, 8, 9, 10, 20, 40, 80, and 150 seeds per pot and measured the number of germinating seeds, the number of individuals that survived up to adulthood, and the total number of open flowers produced by each plant in a lifetime. Their results suggest that, while germination probability is not density‐dependent, an increase in seed density has a negative impact in both probability of plant survival and flower production, an effect attributed by the authors to intraspecific competition. Since these two were also taken as proxies for plant fitness, this means that a change in seed output is not directly proportional to a change in individual plant fitness, so the former cannot be an adequate proxy for the latter. This finding suggests, as the authors point out, that the positive effect provided by the parasitoid to the plant *may be less beneficial for plant fitness than estimated from seed output alone*. This idea seems quite reasonable when taking into account that *S. latifolia* produce a lot of seeds dispersed in a small radius, so that, in the absence of seed predation, seed density could be high enough to decrease the probability of plant survival and amount of flowers produced. The work presented by the authors shows careful experimentation and we believe that it will improve our knowledge of these complex systems, but we do not share some of their conclusions, as explained below.

## 2 SURVIVAL PROBABILITY VERSUS NUMBER OF SURVIVORS

Our greatest reserve is toward figure 5b in the original paper (Villacañas de Castro & Hoffmeister, 2020) and the conclusions derived from it. We recognize that the authors make a good point of making a graph to compare the presence of density‐dependent effects on the probability of germination and survival of plants, but we claim that their choice of what to graph was erroneous for their main purpose. This figure highlights that, as the density of congeners increases, the probability of germination remains almost constant, but the probability of survival of the resulting plants decreases. As the authors state: *Inevitably, this leads us to question what benefit the increase in seed output seen in plants due to parasitism by parasitoid B. variator provides to individual plant fitness*. Nevertheless, we believe that in order to really understand the role of third organisms in balancing the costs and benefits of pollinating seed predators, the focus should not be placed on their impact on individual plant fitness, as measured from their parameters of choice (survival probability and flower production), but rather on their net effect on the plant population performance. Following this line of thought, to explore the final consequences of this density‐dependent process on the dynamics of the plant population size, we think that it might be more suitable to consider the number of individuals that survive, rather than the probability of individual survival. Our rationale is the following: wild populations affected by density‐dependent processes are well known to either tend toward an equilibrium in population size, from above or below, or fluctuate around this theoretical carrying capacity over time (Case , 2003; Murray, 2003; Turchin, 2003). Under this view, and taking into consideration the unspecialized seed dispersal mechanism of *S. latifolia*, we hypothesize that the fact that the survival probability decreases for high seed densities should be expected because the plant must have attained its carrying capacity somewhere along the continuum of densities tested. In fact, this point of view might also be helpful to understand the dependence of reproductive success of the plant population (estimated by flower production per pot) on initial seed densities.

**Figure 1:**
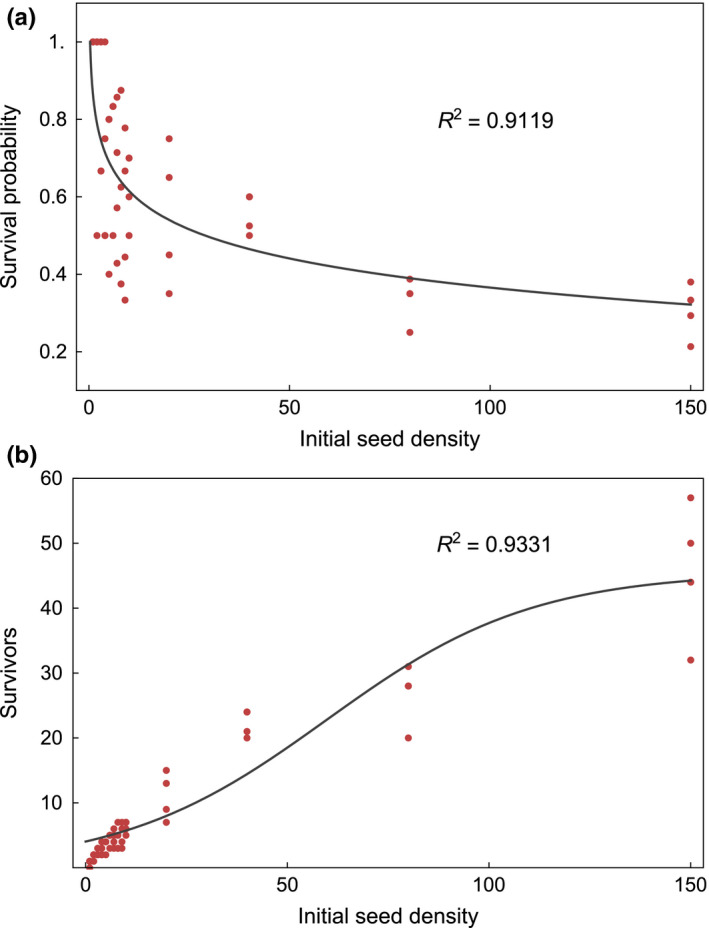
(a): Initial seed density versus survival probability. The original data, shared with us by the authors, were used to replicate here their figure 5b. A fit to a logarithmic function *y* = *a* + *b*log*x* is shown. We obtain coeff. *a* = 0.86, SE = 0.053, *t*‐value = 16, *p*‐value = 1.1 × 10^−22^; coeff. *b* = −0.11, SE = 0.020, *t*‐value = −5.4, *p*‐value = 1.8 × 10^−6^; *R* ^2^ = 0.9119. (b): Initial seed density versus Number of survivors. The same data was used to obtain the number of survivors, by multiplying the initial seed density by the survival probability. By considering the total number of individuals, it is clear that the plant population is not reduced by the presence of parasitoids, as the decrease in survival probability might suggest; on the contrary, it increases. A best fit to a sigmoid function *y* = 1/(*a* + *e* ^−*bx* + *c*^) is shown. We obtain coeff. *a* = 0.022, SE = 0.0013, *t*‐value = 17, *p* ‐value = 2.2 × 10^−23^; coeff. *b* =0.039, SE = 0.0042, *t*‐value =9.2, *p*‐value = 1.4 × 10^−12^; coeff. *c* = −1.5, SE = 0.16, *t*‐value = −9.6, *p*‐value = 3.6 × 10^−13^; *R* ^2^ = 0.9331. The fits were obtained using NonlinearModelFit in Mathematica (Wolfram Research, Inc., 2019) with default starting values

**Figure 2:**
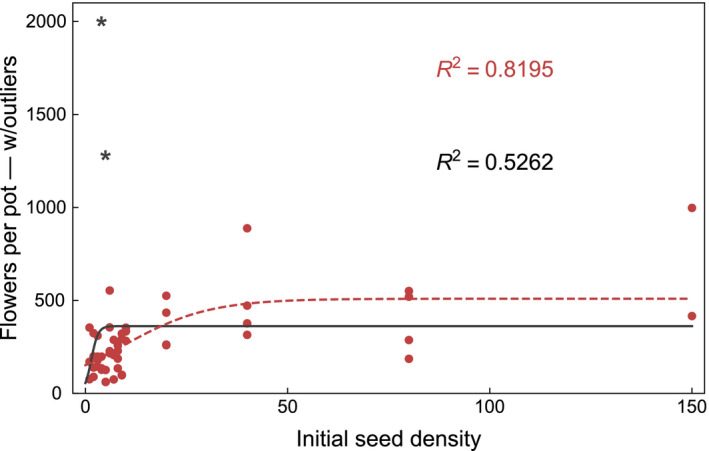
Initial seed density versus mean number of flowers per pot. The mean value of flowers produced per pot during the lifetime of each plant in that pot was calculated by multiplying the mean number of flowers per plant in each pot by the number of survivors in that pot. Two fitting curves, both to a sigmoid function *y* = 1/(*a* + *e* ^−*bx* + *c*^) are shown. For the first one (solid line in black), all data points were considered. We obtain coeff. *a* = 0.0028, SE = 0.00042, *t*‐value = 6.6, *p*‐value = 2.7 × 10^−8^; coeff. *b* = −1.2, SE = 1.9, *t*‐value = 0.60, *p*‐value = 0.55; coeff. *c* = −4.2, SE = 3.7, *t*‐value = −1.1, *p*‐value = 0.26; *R* ^2^ = 0.5262. For the second one (dashed line in red), the two starred (*) black data points were taken to be outliers and were omitted, that is, only the red data points were considered for this fit. We obtain coeff. *a* = 0.0020, SE = 0.00021, *t*‐value = 9.3, *p*‐value = 2.8 × 10^−12^; coeff. *b* = 0.094, SE = 0.044, *t*‐value = 2.1, *p*‐value = 0.040; coeff. *c* = −5.4, SE = 0.34, *t*‐value = −16, *p*‐value = 7.6; *R* ^2^ = 0.8195. The fits were obtained using NonlinearModelFit in Mathematica (Wolfram Research, Inc., 2019) with default starting values

To illustrate these ideas, we present three plots. We used the original data, which Villacanas gently shared with us. In Figure 1a, we plot the initial seed density versus the survival probability of seeds together with a best fitting curve to a logarithmic function, that is, we replicate figure 5b. In Figure 1b, we present the same data, but we plot the number of survivors instead of the probability of survival. This can be calculated by multiplying the initial seed density by the survival probability. A fit to a sigmoid function is included. In Figure 2, we present the mean number of flowers produced per plant versus the initial seed density. We calculated the mean number of flowers measured per plant for each pot and multiplied it by the number of survivors in the same pot, thus obtaining the mean number of flowers produced per pot. We did not use the total number of flowers per plant because not all plants were sampled for flower production. Two fitting curves, both to a sigmoid function, are included. For the first one (solid line in black), all data points were considered. For the second one (dashed line in red), two data points (black, starred) taken to be outliers were omitted, that is, only the red data points were considered for this fit. Although the latter fit is considerably better, a convergence of flower production with increasing initial seed densities can be seen in both. We would expect that, looking only at high initial seed densities (≥20), a clearer convergence should be appreciated. This might be a subject of further scrutiny.

As it can be seen, the plots resemble the classic dynamics of a population that tends toward its carrying capacity from below, giving credibility to our hypothesis. This shows that the plant population size would not be limited by the presence of parasitoids, as the decrease in survival probability might suggest; on the contrary, as long as the plant population size is below its carrying capacity, it increases in size in their presence. This suggests that the parasitoid acts as a mutualist for the plant (reducing the herbivore pressure), but only up to a certain threshold, determined by the carrying capacity of the plant populations. After this threshold is reached, further increases in seed output will not increase the plant population size. It is important to consider that, for this interpretation, we are assuming that plant populations do not overlap, that is, that there is no delay in their dynamics. In a population model without time delays, the intrinsic growth ratio is a fixed value. Even when a population's reproductive success is ultimately determined by its effective growth ratio, this parameter depends on the present value of the population and its relative value to its carrying capacity. We now present a theoretical analysis that will help us define under which precise conditions the parasitoids act as mutualists for the plants and hopefully also better explain this last point.

## 3 THEORETICAL ANALYSIS

Another point that we would like to discuss is that the authors hypothesize that, at higher plant densities, survival and flower production would decrease due to intraspecific competition and that this might diminish or reverse the positive effect due to the presence of the parasitoid. In our opinion, from a population dynamics perspective, the strength of intraspecific competition could easily increase or decrease the magnitude of the change in plant population size when the parasitoid is present, but the sign of this change (i.e., whether the population size increases or decreases) is determined mainly by the interspecific interactions, instead of the intraspecific ones. To show this, we present here a theoretical analysis based on a population dynamics model that we recently developed to simulate this tritrophic system (Stucchi et al., 2019), in which we demonstrated that the system is more stable with the presence of parasitoids. We think this model may also help us clarify the role of plant intraspecific competition on the stability of the system in the presence of parasitoids.

In our equations (Equations (2), (3), (4), (5), (6) in Stucchi et al. (2019)), plant population is represented by $X_{1}$, male and female moths are represented by $X_{2}$ and $X_{3}$, respectively, and parasitoids are represented by $X_{4}$. The sexes of moths were modeled separately in order to account for their antagonistic–mutualistic roles. Thus, the female moths were assumed to be purely antagonistic in relation to the plants, they being the ones to lay eggs whose larvae predate on the plants seeds, while the male moths were assumed to be genuine mutualists (pollinators). Although female moths also contribute to pollination, this simplification made it simpler to estimate values for the parameters in our equations from available data. We are interested in Equation 2 of Stucchi et al. (2019), which reads:

(1) $$\frac{{\dot{X}}_{1}}{X_{1}}=\left(r_{1}+b_{12}X_{2}+b_{13}X_{3}\right)‐\left(a_{1}+c_{1}\left(b_{12}X_{2}+b_{13}X_{3}\right)\right)X_{1}.$$

Here, $r_{1}\in R$represents the intrinsic growth rate of population $X_{1}$, that is, of *S. latifolia*. The parameters $b_{1j}$ represent the interaction between the plants and population $X_{j}$, so $b_{12}>0$ represents the beneficial effects of male moths on the plants and $b_{13}<0$ represents the negative effects of female moths on the plants. Lastly, both $a_{1}>0$ and $c_{1}>0$ represent saturation effects on the plant: $a_{1}$, which we call *saturation rate*, is caused by intraspecific competition for environmental resources, while $c_{1}$ represents the saturation caused by the occupation rate by members of the same population and which ultimately limits their intraspecific interactions. The signs for these parameters were deduced in Stucchi et al. (2019).

To describe the equilibrium point of the plant population, $X_{1}$, we do:

(2) $$0={\dot{X}}_{1}=X_{1}\left(r_{1}‐a_{1}X_{1}+b_{12}X_{2}\left(1‐c_{1}X_{1}\right)‐b_{13}X_{3}\left(1‐c_{1}X_{1}\right)\right),$$

where, for convenience, we have set $b_{13}>0$ and changed sign appropriately (so $b_{13}$ in Equation 1 → $‐b_{13}$ in Equation 2).

Let's assume that there is a small perturbation in the parasitoid population, that is, that some amount $\delta X_{4}$ of parasitoids suddenly appears in the system. This will produce perturbations in the male and female moth populations, respectively, $\delta X_{2}$ and $\delta X_{3}$. After adding in our perturbations, the nontrivial equilibrium point will be given by

(3) $$\begin{array}{c} 0=\left(X_{1}+\delta X_{1}\right)(r_{1}‐a_{1}\left(X_{1}+\delta X_{1}\right)+b_{12}\left(X_{2}‐\delta X_{2}\right)\left(1‐c_{1}\left(X_{1}+\delta X_{1}\right)\right)\\ ‐b_{13}\left(X_{3}‐\delta X_{3}\right)\left(1‐c_{1}\left(X_{1}+\delta X_{1}\right)\right)), \end{array}$$

where we have chosen the signs in front of $\delta X_{2}$ and $\delta X_{3}$ to be negative because an increase in the parasitoid population will cause a decrease in the overall moth population, affecting both males and females. Now, we are interested in the resulting perturbation in the plant population after the introduction of the parasitoids, so we solve Equation (3), (4) for $\delta X_{1}$, obtaining

(4) $$\delta X_{1}=\frac{\left(b_{13}\delta X_{3}‐b_{12}\delta X_{2}\right)\left(1‐c_{1}X_{1}\right)}{a_{1}+c_{1}\left(\left(b_{13}\delta X_{3}‐b_{12}\delta X_{2}\right)+\left(b_{12}X_{2}‐b_{13}X_{3}\right)\right)}.$$

An assumption in Stucchi et al. (2019) is that $1/c_{1}$ is an upper bound for the population size $X_{1}$, that is, that $c_{1}X_{1}<1$. Because of this assumption, the sign of $\delta X_{1}$ depends on the values of $a_{1}$, $\left(b_{13}\delta X_{3}‐b_{12}\delta X_{2}\right)$, and $\left(b_{12}X_{2}‐b_{13}X_{3}\right)$. The sign of $\delta X_{1}$ might change, given a fixed $a_{1}$, if the ratios $\left(b_{12}\delta X_{2}/b_{13}\delta X_{3}\right)$ or $\left(b_{12}X_{2}/b_{13}X_{3}\right)$ change. This means that the net change on the plant population is determined by the ratio between the current populations of male and female moths, the ratio between the changes in these populations, and the ratio between the interspecific interaction parameters $b_{1j}$.

However, there is another possibility. Given certain values of $b_{1j},X_{2},X_{3},\delta X_{2}$, and $\delta X_{3}$ the sign of $\delta X_{1}$ might also change if $a_{1}$, the parameter we associated with intraspecific competition, changes by an amount $\delta a_{1}$ and

(5) $$\left|a_{1}+c_{1}\left(\left(b_{13}\delta X_{3}‐b_{12}\delta X_{2}\right)+\left(b_{12}X_{2}‐b_{13}X_{3}\right)\right)\right|<\left|\delta a_{1}\right|,$$

given that both sides of the inequality would have different signs. It is interesting to note that the latter possibility depends strongly on the value of $c_{1}$. According to Stucchi et al. (2019), $c_{i}$ measures the “relative presence of individuals of a species in the area under study” which ultimately reflects the interspecific competition for trophic interactions. In this case, $c_{1}$ measures how the presence of other plants might saturate the effects that male and female moths would cause on *Silene latifolia*. If we neglect this saturation, by considering $c_{1}=0$, we obtain

(6) $$\delta X_{1}=\frac{b_{13}\delta X_{3}‐b_{12}\delta X_{2}}{a_{1}}.$$

In this approximation, $\delta X_{1}$ will be positive, negative, or zero depending on the ratio $\left(b_{13}\delta X_{3}/b_{12}\delta X_{2}\right)$, but the resulting sign does not depend on $a_{1}$. In other words, the magnitude of change experienced by the plant population size after the introduction of parasitoids partially depends on the intraspecific competition between plants, but the sign of this change depends only on the interaction between plants and male and female moths. Consequently, the magnitude of the intraspecific competition between plants only plays an essential role in the system when the interspecific saturation, $c_{1}$, is considered.

## 4 CONCLUDING REMARKS

The work presented by Villacañas shows careful experimentation and constitutes a valuable effort to provide new data on this tritrophic interaction system. We do share some of their conclusions, such as that seed output per plant is not a good proxy for plant lifetime fitness. However, we claim that they do not successfully address whether the presence of the parasitoid modifies the interaction between the plant and the pollinating seed predator along the mutualism–antagonism gradient. Our analysis suggests that this question is better investigated by focusing not on individual fitness, but rather on the effects on population size, which we refer to as population performance. In particular, by focusing on the number of survivors up to adulthood (i.e., the initial seed density multiplied by the survival probability, in the authors' terms), we were able to address the net impact of the parasitoid on the plant. We show this in Figure 2, whose graph resembles the dynamics of a population that tends toward its carrying capacity from below. This shows that the increase in seed density provided by the parasitoid (by reducing the level of seed predation) is beneficial to the plant population until its carrying capacity is achieved.

Furthermore, the authors hypothesize that, at higher plant densities, survival and flower production would decrease due to intraspecific competition, and hence that it is this competition what can diminish or reverse the positive effect of the presence of the parasitoid to the plant. We have presented an analysis based on a population dynamics model, showing which particular conditions would be necessary for intraspecific competition to be more detrimental to the plant than its increase in seed output is beneficial. In particular, when ignoring our $c_{1}$ term, we find that, in the presence of the parasitoids, a change in the magnitude of plant intraspecific competition may either increase or decrease the magnitude of plant population change, but not the sign of this change (i.e., whether the population size increases or decreases), which is only conditioned by the interspecific interactions. Therefore, the role of plant intraspecific competition is limited to the prevention of unbounded population growth in the presence of parasitoids, here acting as mutualists for the plant. Indeed, we have already shown that the introduction of a parasitoid species increases the equilibrium density of the plant above its carrying capacity when interacting only with the nursery pollinator (Stucchi et al., 2019). These results are in agreement with many studies on the population ecology of mutualistic organisms, which show that several intrinsic and extrinsic factors, intra‐ and interspecific competition among others, may contribute to the stability of mutualisms (Holland et al., 2002; Thompson, 2005).

## CONFLICTS OF INTERESTS

None declared.

## AUTHOR CONTRIBUTIONS

LGB initiated the collaborative commentary. NMG, LS, and JG performed the formal analysis. All authors contributed to the drafts and final version.

## Data Availability

Original dataset from Villacañas de Castro & Hoffmeister (2020) Dryad:doi.org/10.5061/dryad.rjdfn2z75.
